# Supplementary material for: A Fkh1/2 binding site array in the WHI5 promoter drives sub-scaling transcription
Source: Cell Rep. Author manuscript; Available in PMC 2026 Jun 23. (PMC13288135; doi:10.1016/j.celrep.2026.117304)
Supplement: 1 [file NIHMS2180947-supplement-1.pdf]

Cell Reports, Volume *45*

Supplemental information

A Fkh1/2 binding site array in the *WHI5* promoter drives sub-scaling transcription

Jacob Kim, Shicong Xie, Lucas Fuentes Valenzuela, Jordan Xiao, Mike C. Lanz, Xin Gao, Matthew Swaffer, Corbin E. Mitchell, Seth M. Rubin, Kurt Schmoller, and Jan M. Skotheim

SUPPLEMENTAL FIGURES

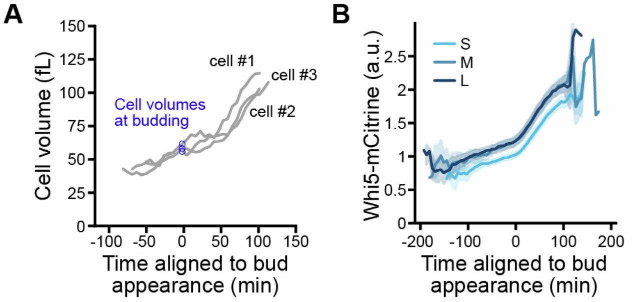

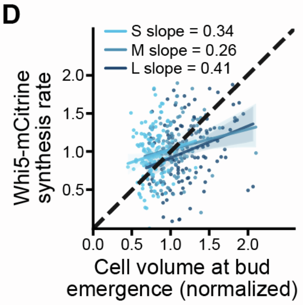

E

Whi5-mCitrine synthesis rate ~ $\beta_1 x_1 + \beta_2 x_2 + \beta_3 x_3 + \beta_4$

Variable (*x*)	Coefficient w/ 95% confidence intervals (*β*)	p-value
normalized volume at bud emergence (x_1)	0.36 ± 0.15 (β_1)	<0.01
+5ng/µL aTc (x_2)	-0.01 ± 0.01 (β_2)	0.82
+30ng/µL aTc (x_3)	-0.10 ± 0.10 (β_3)	0.06
constant	0.68 ± 0.13 (β_4)	<0.01

Figure S1: Exogenous Whi5 does not impact expression from the endogenous *WHI5* promoter. Related to Figure 1.

(A) Cell volume dynamics for the corresponding cells shown in **Figure 1D**.

(B) Average Whi5-mCitrine total fluorescence intensity dynamics aligned at budding. Small, medium, and large cells express increasing amounts of exogenous, untagged Whi5 as shown in **Figures 1F-G**. Shaded region denotes 95% confidence intervals. (small: n=140, medium: n=111, large: n=120)

(C) Average cell volume dynamics, and 95% confidence intervals, for the same sets of cells shown in panel B.

(D) Whi5-mCitrine synthesis rates normalized to the average rate and plotted as a function of cell size at bud emergence, which was normalized to the mean. Individual data points shown for Whi5-mCitrine synthesis rates corresponding to the binned averages shown in **Figure 1H**. The shaded regions denote the 95% confidence intervals of linear fits. (small: n=140, medium: n=111, large: n=120)

(E) Multivariate linear regression statistics that did not identify a significant effect of exogenous Whi5 (medium and large cell size variables) on the synthesis rate of the endogenous Whi5-mCitrine.

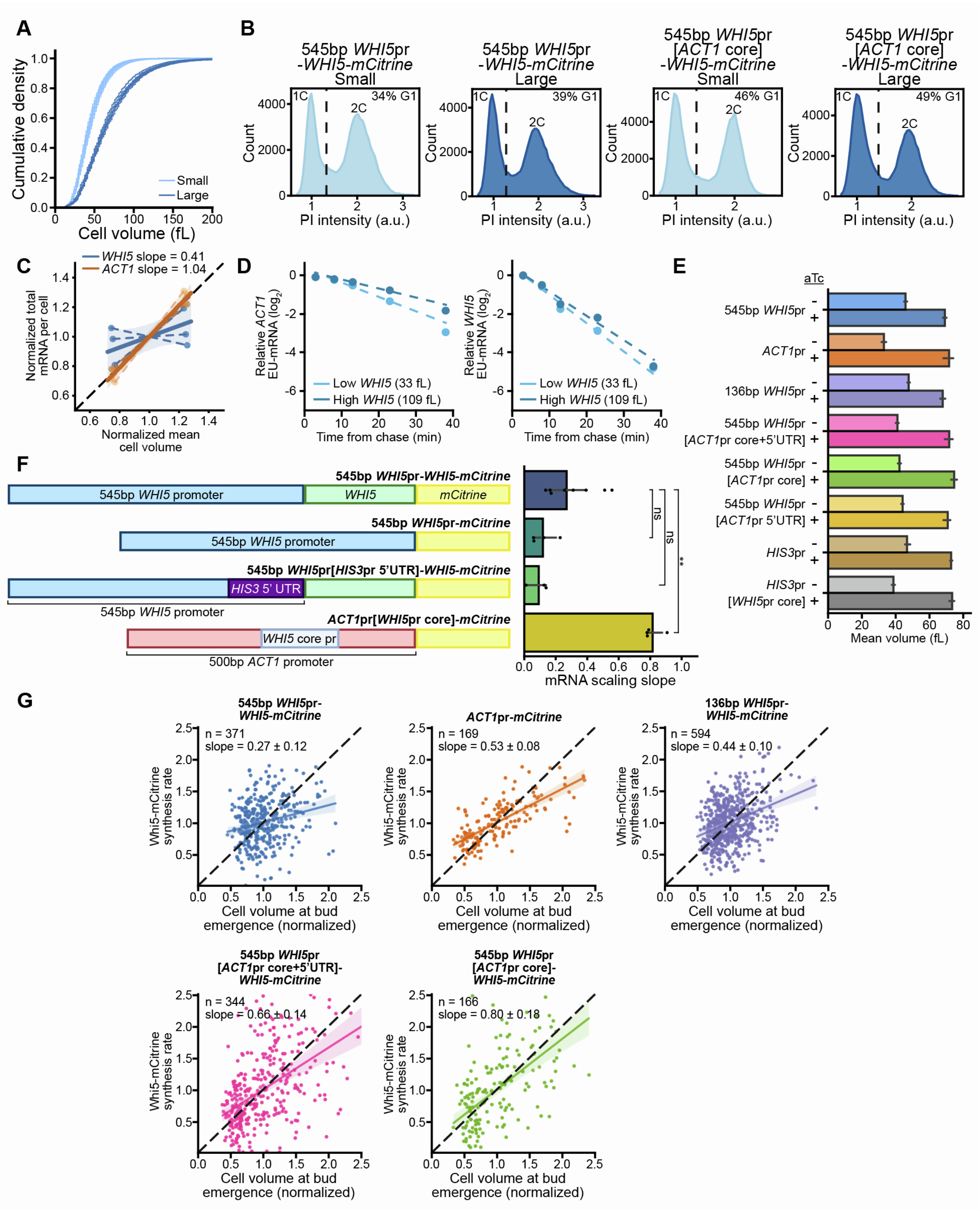

Figure S2: RT-qPCR analysis of cell size scaling and corroboration by single cell imaging data. Related to Figure 2.

(A) Cumulative distributions of cell size measured using a Coulter counter for 9 replicates of cells exposed to 0 or 30ng/mL aTc.

(B) Flow cytometry data of propidium iodide (PI) stained yeast cells for the indicated genotypes showing the distribution of DNA content used to estimate the fraction of cells in G1 phase of the cell cycle (see **methods**).

(C) Spike-in normalized RNA-seq data from Swaffer et al. 2023 [1] plotted to show *WHI5* and *ACT1* mRNA per cell for wild-type and larger *cln3*Δ cells. Black dashed line represents perfect size scaling where slope=1. For each gene, the mRNA values were normalized to the average of the small and large cells in each experiment. Dashed lines represent the scaling slope from each experiment, while solid lines represent a linear regression calculated from all experiments for each gene.

(D) Data from Swaffer et al. 2023 [1] showing the degradation of *WHI5* and *ACT1* mRNA. A spike-in normalized EU pulse-chase experiment was used to determine mRNA turnover rates in cells with low (33fL) or high (109fL) exogenous *WHI5* expression from a beta-estradiol responsive promoter. Time is aligned to EU washout (chase) following a 1-hour EU pulse. The mean (± range) of two biological replicates is shown. Dashed lines show exponential fits to the data.

(E) Mean cell volumes used to calculate size scaling slopes in **Figure 2E**. Mean values were calculated from cell volume distributions measured by a Beckman Coulter Z2 Cell and Particle Counter. (545bp *WHI5*pr: n=9, *ACT1*pr: n=4, 136bp *WHI5*pr: n=8, 545bp *WHI5*pr[*ACT1*pr core+5'UTR]: n=5, 545bp *WHI5*pr[*ACT1*pr core]: n=5, 545bp *WHI5*pr[5'UTR]: n=4, *HIS3*pr: n=9, *HIS3*pr[*WHI5*pr core]: n=9)

(F) Schematic representation of the promoters whose expression is examined. The core *ACT1* promoter was replaced by the core *WHI5* promoter in one strain, while the *WHI5 5'UTR* was replaced with the corresponding region of *HIS3* in another strain. Right hand side: Size scaling slope calculated as shown in **Figures 2A and 2B** for the indicated promoters. Error bars indicate the standard deviation, and each data point represents a separate biological replicate. For the indicated comparisons, ns denotes p>0.05 and ** p< 0.01. 545bp *WHI5*pr-*WHI5-mCitrine* data are reproduced from 545bp *WHI5*pr from **Figure 2E**. (545bp *WHI5*pr-*WHI5-mCitrine*: n=9, 545bp *WHI5*pr-*mCitrine*: n=4, 545bp *WHI5*pr[*HIS3*pr 5'UTR]-*WHI5-mCitrine*: n=4, *ACT1*pr[*WHI5*pr core]-*mCitrine*: n=4)

(G) Whi5-mCitrine synthesis rates calculated as shown in **Figure 1D** plotted against the cell size at bud emergence. Each panel corresponds to the indicated genotype and data points correspond to single cells. The linear regression and associated 95% confidence intervals are shown. Black dashed lines represent perfect size scaling where slope = 1. Data summary is shown in **Figure 2G**. (545bp *WHI5*pr: n=371, *ACT1*pr: n=169, 136bp *WHI5*pr: n=594, 545bp *WHI5*pr[*ACT1*pr core+5'UTR]: n=344, 545bp *WHI5*pr[*ACT1*pr core]: n=166)

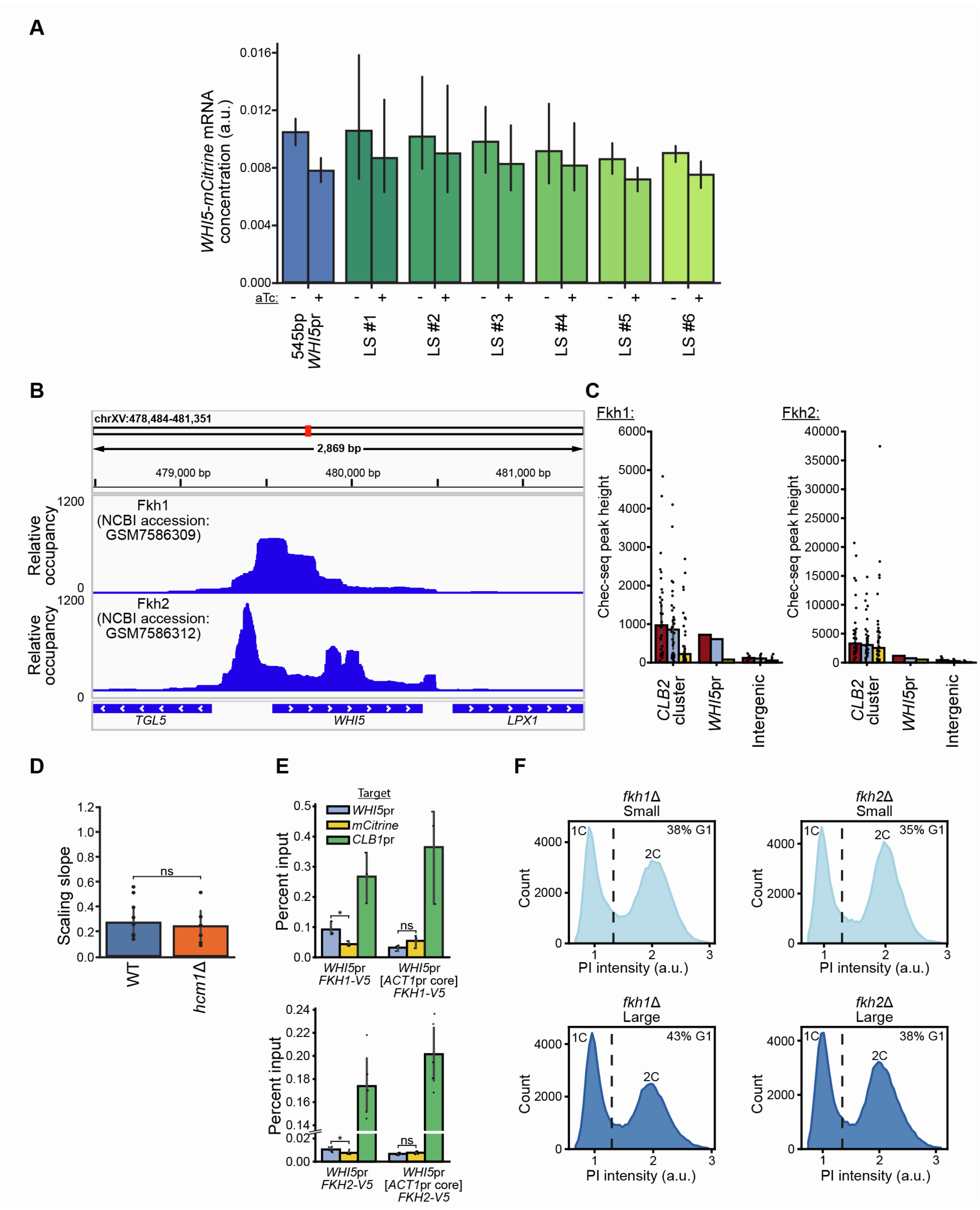

Figure S3: Data supporting the association of Fkh1 and Fkh2 with the *WHI5* promoter. Related to Figure 3.

(A) mRNA concentrations expressed from the indicated LS mutants shown in **Figure 3A** in large and small populations of cells (±30ng/mL aTc). mRNA concentrations were measured relative to the

endogenous *ACT1* mRNA.

(B) Fkh1 and Fkh2 ChEC-sequencing data from Mahendrawada et al. 2025 [2] showing enrichment of DNA from the *WHI5* promoter region. ChEC-seq (Chromatin Endogenous Cleavage followed by sequencing) is a genomic technique that maps where a chromatin-associated protein sits on DNA similar to the more conventional ChIP-seq technique. Bigwig files for Fkh1 and Fkh2 (GEO GSE236948) were aligned to the *S.cereviasiae* genome and visualized using Integrative Genomics Viewer (IGV).

(C) ChEC-seq peak heights of Fkh1 and Fkh2 from Mahendrawada et al. 2025 [2] at Fkh-regulated *CLB2* cluster promoters, the *WHI5* promoter, and background intergenic regions. The *CLB2* cluster genes were defined from Spellman et al 1998 [3]. The intergenic regions chosen were the 6 regions flanking *WHI5* (3 upstream, 3 downstream) between genes positioned in opposite directions. The error bars indicate the 95% confidence interval of the median calculated from bootstrapping. The 3 colored bars are from the 3 data sets of independent biological replicates.

(D) Size-scaling slopes for mRNA expression calculated as in **Figure 2B** shown for WT and *hcm1*Δ backgrounds. For the indicated comparison, ns denotes p>0.05. WT data are reproduced from 545bp *WHI5*pr from **Figure 2E**. (WT: n=9, *hcm1*Δ: n=6)

(E) Chromatin immunoprecipitation followed by RT-qPCR performed for the indicated strains, in which the *FKH1* or *FKH2* gene has been fused to a V5 tag. qPCR primers target the *WHI5* core promoter region, mCitrine, or the *CLB1* promoter. Error bars indicate 95% confidence intervals, and each data point represents a separate biological replicate (*FKH1-V5* n=3, *FKH2-V5* n=5).

(F) Flow cytometry data of propidium iodide (PI) stained yeast cells for the indicated genotypes showing the distribution of DNA content used to estimate the fraction of cells in G1 phase of the cell cycle (see **methods**).

All statistical tests performed were Student's t-tests.

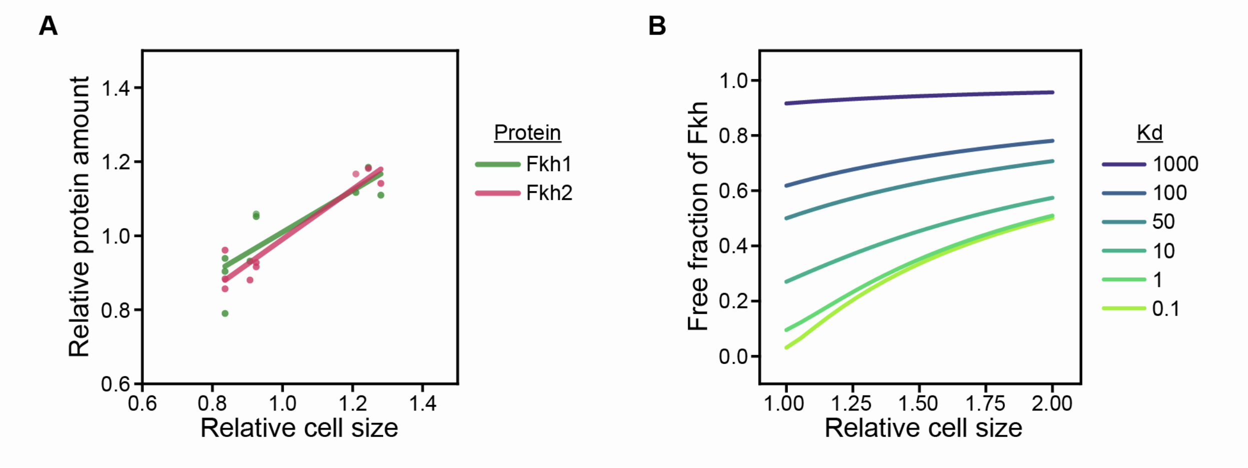

Figure S4: The concentration of free Fkh increases with cell size. Related to Figure 4.

(A) Mass spectrometry measurements of relative protein amounts in differently-sized, *whi5*Δ, wildtype, and *cln3*Δ strains from Lanz et al 2024 [4]. (n=3 for each strain and protein).

(B) While the concentration of total Fkh (C_0) remains constant, cell size increases the concentration of free Fkh because a lower proportion of the transcription factor is bound to the sites on the genome. This is due to the dilution of the fixed number of nuclear Fkh binding sites (N_s) as the nucleus grows larger. Concentration of unbound Fkh as a function of cell volume for different values of the dissociation constant K_d. The parameters used are C_0=100nM and N_s=500.

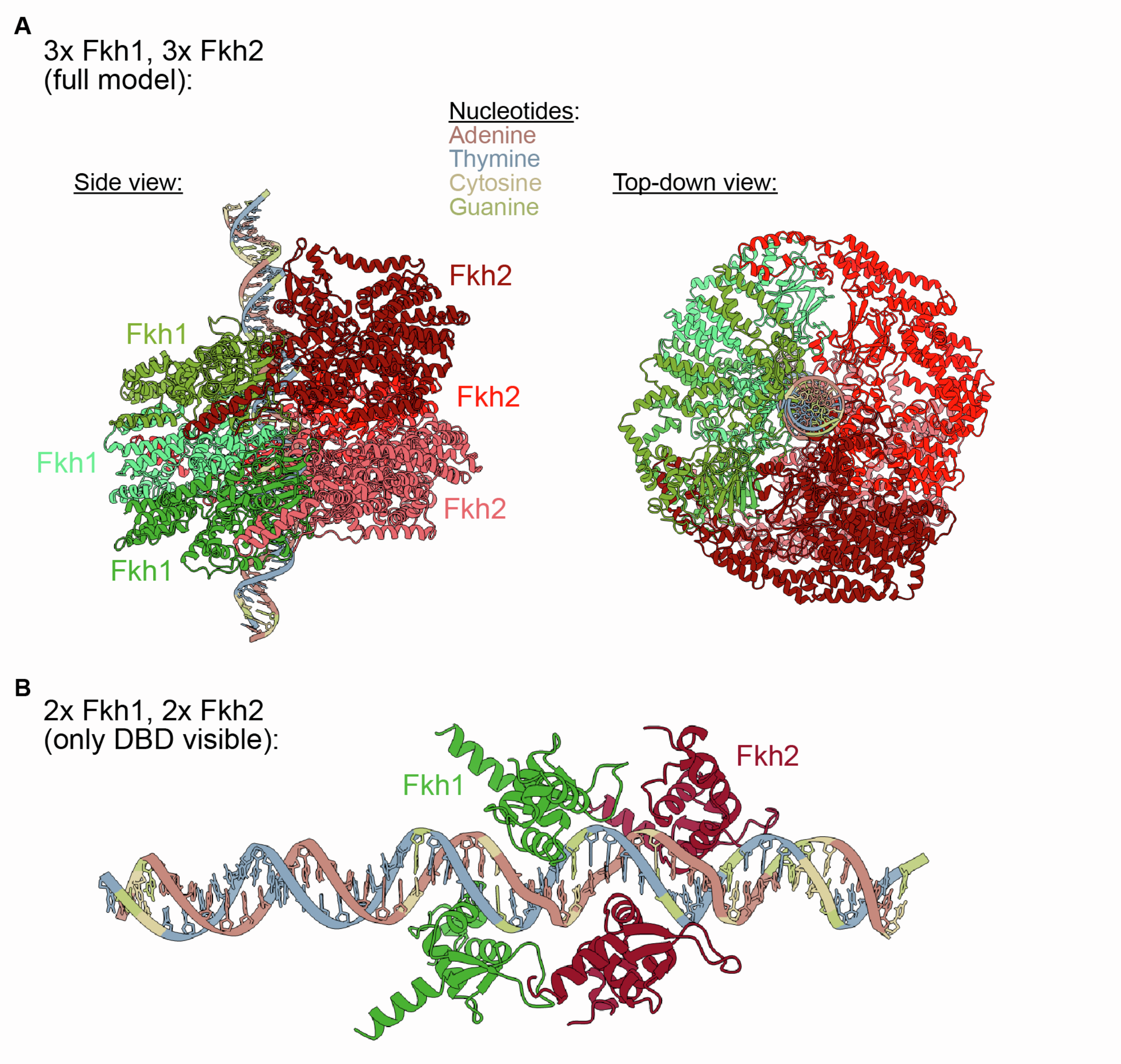

Figure S5: AlphaFold3 structural predictions showing Fkh1 and Fkh2 binding the core *WHI5* promoter. Related to Figure 5.

(A) AlphaFold3 structural model of 3 Fkh1 proteins, 3 Fkh2 proteins, and the 51 bp *WHI5* core promoter. Fkh1 and Fkh2 molecules wrap around the core promoter, forming a multimer along each Fkh binding motif. This model displays Fkh1 and Fkh2 molecules on opposite sides of the DNA. Left: viewed laterally from DNA. Right: viewed down the double helix structure.

(B) DNA binding domains (DBD) of 2 Fkh1 and 2 Fkh2 proteins bound to the core *WHI5* promoter. An alternate model showing Fkh1 and Fkh2 occupying Fkh binding sites on both sides of the DNA.

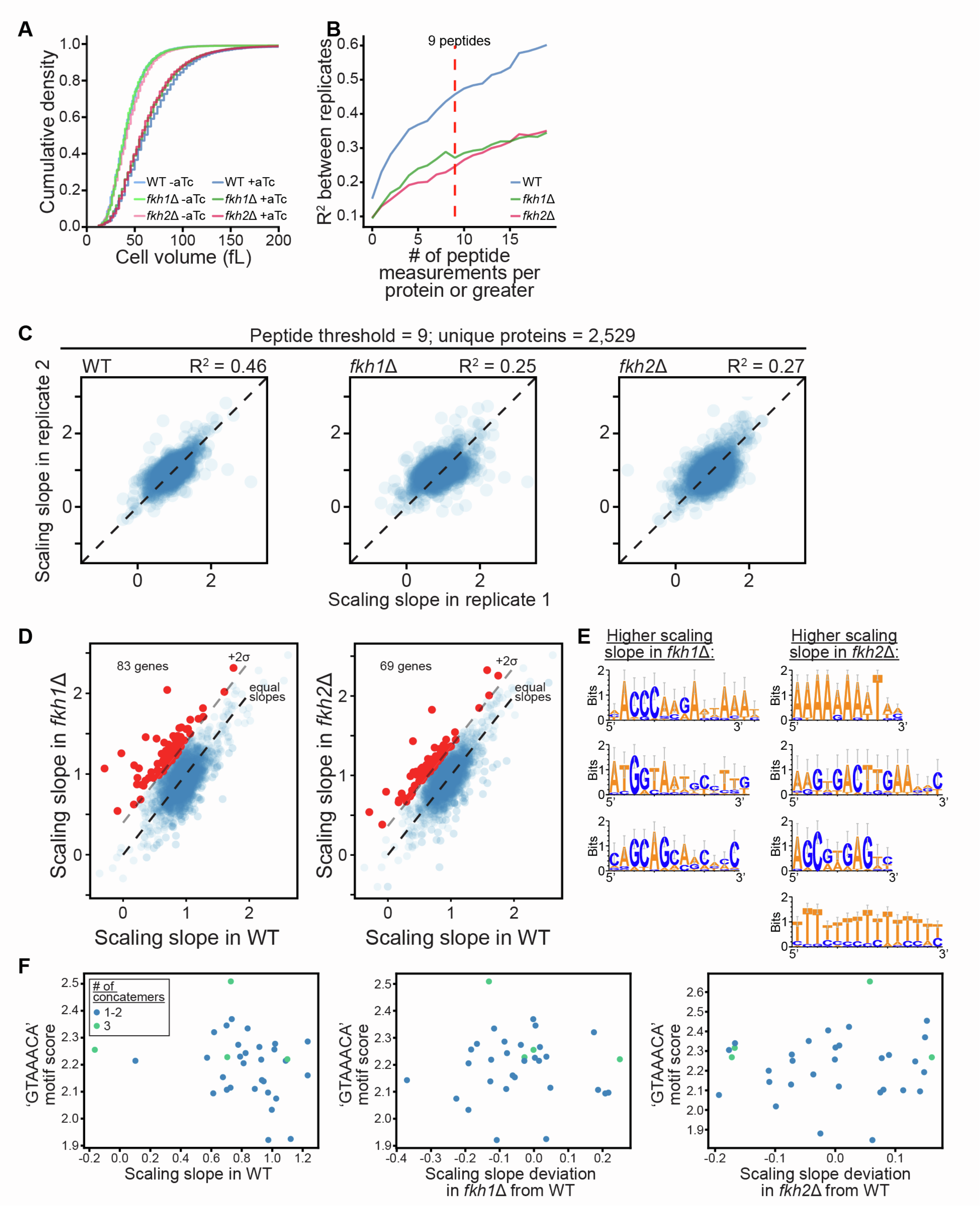

Figure S6: Proteomic and bioinformatic analysis of size scaling in *fkh1*Δ and *fkh2*Δ cells. Related to Figure 6.

(A) Cumulative probability distributions of cell size measured using a Coulter counter for cells of the indicated genotype exposed to 0 or 30ng/mL aTc, which controls the expression of an exogenous *WHI5*

allele to generate population of small and large cells, respectively.

(B) The size scaling slope was measured for proteins using mass spectrometry data as described for **Figure 6B**. Slopes were compared between experimental replicates to calculate the coefficient of determination, R^2, when only proteins for which the indicated minimum number of peptides were included. The stricter this criterion, the higher the correlation between replicate experiments, but the lower the number of proteins that were measured. To balance this tradeoff, we set this peptide threshold to 9 (red dashed line).

(C) Replicate correlation of scaling slope measurements for the indicated genotypes with the peptide threshold=9.

(D) Size scaling slopes calculated from protein amount estimates from the proteomics measurements for *fkh1*∆ and wild-type cells. The relative protein concentrations are taken from the mass spectrometry data and multiplied by the average cell size of the population, measured using a Coulter counter, to get the relative protein amounts. Cell size and protein amounts are normalized by the mean of the small and large cell samples for each strain. Scaling slope = (large cell protein amount - small cell protein amount)/(large cell size - small cell size).

Red dots indicate proteins whose slopes are more than 2 standard deviations above the trend value.

(E) Sequence motifs that were enriched in the group of genes whose slopes increased upon deletion of *fkh1*∆ or *fkh2*∆ (red data points in panel D; see **methods**).

(F) Protein scaling behavior of genes whose promoters contain 2 or 3 canonical ‘GTAAACA’ Fkh motifs. The ‘GTAAACA’ motif score indicates a position-weighted matrix score of ‘GTAAACA’ sites applied to the promoter region. Left: protein scaling behavior in WT background. Middle: scaling slope deviation from WT upon *fkh1Δ.* Right: size-scaling slope deviation from WT upon *fkh2Δ.*

REFERENCES

1. Swaffer, M.P., Marinov, G.K., Zheng, H., Fuentes Valenzuela, L., Tsui, C.Y., Jones, A.W., Greenwood, J., Kundaje, A., Greenleaf, W.J., Reyes-Lamothe, R., et al. (2023). RNA polymerase II dynamics and mRNA stability feedback scale mRNA amounts with cell size. Cell *186*, 5254-5268.e26. https://doi.org/10.1016/j.cell.2023.10.012.

2. Mahendrawada, L., Warfield, L., Donczew, R., and Hahn, S. (2025). Low overlap of transcription factor DNA binding and regulatory targets. Nature *642*, 796–804. https://doi.org/10.1038/s41586-025-08916-0.

3. Spellman, P.T., Sherlock, G., Zhang, M.Q., Iyer, V.R., Anders, K., Eisen, M.B., Brown, P.O., Botstein, D., and Futcher, B. (1998). Comprehensive identification of cell cycle-regulated genes of the yeast Saccharomyces cerevisiae by microarray hybridization. Mol. Biol. Cell *9*, 3273–3297. https://doi.org/10.1091/mbc.9.12.3273.

4. Lanz, M.C., Zhang, S., Swaffer, M.P., Ziv, I., Götz, L.H., Kim, J., McCarthy, F., Jarosz, D.F., Elias, J.E., and Skotheim, J.M. (2024). Genome dilution by cell growth drives starvation-like proteome remodeling in mammalian and yeast cells. Nat. Struct. Mol. Biol. *31*, 1859–1871. https://doi.org/10.1038/s41594-024-01353-z.
